# A Bionic Multichannel Whisker System for Assisting Endoluminal Intervention

**DOI:** 10.34133/cbsystems.0616

**Published:** 2026-08-05

**Authors:** Zeyu Wang, Frank P.-W. Lo, Junhong Chen, Jianing Qiu, Qian Chen, Yongqi Zhang, Chen Lin, Kyle Lam, James Calo, Benny P. L. Lo, Alexander J. Thompson, Eric M. Yeatman

**Affiliations:** ^1^ Department of Surgery and Cancer, Faculty of Medicine, Imperial College London, London SW7 2AZ, UK.; ^2^ Department of Electrical and Electronic Engineering, Faculty of Engineering, Imperial College London, London SW7 2AZ, UK.; ^3^ Department of Personalized Medicine, Mohamed bin Zayed University of Artificial Intelligence, Building 1B, Masdar City, P.O. Box 7909, Abu Dhabi, United Arab Emirates.; ^4^ College of Science and Engineering, University of Glasgow, Glasgow G12 8QQ, UK.

## Abstract

Endoluminal interventions are crucial for both the diagnosis and treatment of gastrointestinal diseases. However, conventional endoscopic systems are designed for visual inspection and diagnosis, limiting their capacity to assess critical tissue properties such as texture, stiffness, and structural integrity. These unobserved mechanical signatures may contribute to clinically relevant failure modes, including missed lesions, incomplete resection, and adverse events driven by excessive wall loading. Inspired by the tactile sensing mechanisms of rat whiskers, this study introduces a bionic multichannel whisker system, as an additional sensing modality for endoluminal interventions. The system integrates high-fidelity tactile sensing hardware, and advanced signal processing algorithms, enabling precise and reliable measurements of tissue properties, as well as providing real-time radial force feedback. Calibration via affine transformation ensures cross-channel consistency and compensates for manufacturing and installation variances. Validation across multiple experimental settings, including robotic and manual operation, demonstrates performance in texture discrimination, shape reconstruction, and radial force estimation. The presented whisker system is compact to support integration with endoscopic instruments, providing a practical basis for complementing endoluminal diagnostic workflows and for further development of tactile sensing in surgical robotics.

## Introduction

Endoluminal interventions have fundamentally transformed the diagnosis and treatment of gastrointestinal (GI) diseases. These minimally invasive procedures involve the deployment endoscopes into the luminal organs and enable direct access for visual inspections, tissue biopsy, and therapeutic intervention [1–3]. Their multifaceted benefits, including the reduction of patient trauma, the shortened recovery times, and the improved diagnostic accuracy, are especially vital in combating the substantial global burden of GI disorders, which encompass a wide range of conditions, from inflammatory bowel diseases to colorectal cancer [4–6].

Conventional endoscopic systems form the foundation of endoluminal interventions, providing the essential tools for visualization, diagnosis, and treatment within the GI tract. Endoscopes allow introduction of clinical instruments such as biopsy forceps, snares, and electrosurgical devices, and also provide high-resolution optical imaging that is used for diagnosis via careful inspection of the GI mucosa. Mainstream imaging systems typically incorporate white-light complementary metal-oxide-semiconductor (CMOS) sensors as their core components, with HD (1,920 × 1,080) or 4K (3,840 × 2,160) resolution and a 100° to 170° field of view, integrated with customized light sources to enhance illumination conditions. Recent advancements introduce 4K and even 8K imaging capabilities [7]. Modern endoscopic systems are further enhanced by advanced imaging modalities, such as narrow-band imaging (NBI) [8], confocal laser endomicroscopy [9], and chromoendoscopy [10], which improve the detection and characterization of subtle pathological changes.

Despite the advancements in conventional endoscopic systems, these technologies are still constrained by inherent limitations that impact their diagnostic and therapeutic efficacy. The performance of conventional endoscopic systems is heavily influenced by external factors, including lighting conditions, operator expertise, and the quality of imaging components. Inadequate illumination or suboptimal imaging components can compromise visual clarity, leading to diagnostic inaccuracies, and inconsistencies in procedural outcomes [11,12]. These challenges undermine the reliability of endoscopic examinations, particularly in detecting subtle GI abnormalities [13]. Even with modern imaging adjuncts, tandem colonoscopy studies report substantial adenoma miss rates, indicating persistent limitations of vision-centric screening under real-world conditions [14]. Another major shortcoming is the reliance on visual inspection, which limits access to physical properties such as surface texture, and fine local structural variation that may carry clinically relevant information [15,16]. Subtle mucosal abnormalities, which could be caused by precancerous lesions or inflammatory irregularities, may present as fine surface irregularities, shallow contour changes, or low-profile protrusions that are not always reliably detected under nonideal viewing conditions [17,18]. This challenge is reflected by the continuing development of image-enhanced endoscopic techniques, which aim to improve the visibility of subtle mucosal surface abnormalities and microstructural patterns rather than relying on conventional white-light imaging alone. In parallel, many clinically important colorectal lesions occur in the diminutive to small size range, and superficially elevated lesions may differ from polypoid lesions by only a few millimeters in height, further motivating complementary sensing approaches that can resolve local geometric deviation at clinically meaningful scale.

Navigating the endoscope within confined or tortuous anatomical regions also poses substantial challenges. The absence of additional sensory feedback mechanisms increases the risk of procedural complications, such as looping, tissue perforation, or misjudgment of tissue proximity [19–21]. Clinically, loop formation and over-angulation can increase patient discomfort and may elevate perforation risk in vulnerable anatomy, including diverticular disease and postsurgical adhesions [22]. Visual information alone may be insufficient for guiding precise movements in such environments, particularly when tissue properties or spatial constraints obscure the endoscope trajectory. To address these challenges, the integration of real-time radial force feedback has emerged as a promising complementary strategy [23–26]. Solutions such as magnetic endoscope imaging can support loop recognition and scope straightening, but they do not quantify local wall loading at the instrument–tissue interface [27]. By providing tactile information about tissue interaction, force-sensing technologies could improve navigation safety and precision, especially during early contact, sustained wall loading, or collision-prone maneuvers. They may also support complementary assessment of tissue stiffness, which is often associated with pathological change [28,29]. These considerations motivate sensing approaches that can capture radial contact information in confined endoluminal environments and support safer instrument–tissue interaction.

Recent investigations into rat whiskers have highlighted the potential of whisker-based tactile sensing for addressing these limitations in endoscopic technologies, particularly in clinical applications requiring enhanced navigation and tissue characterization [30–32]. Given low retinal cell density and fewer cone cells in the rat visual system, whisker-mediated tactile sensing of rat plays a central role in texture perception, distance estimation, and navigation in confined and poorly illuminated environments. This biological adaptation provides a promising model for endoscopic systems, which often face similar challenges when navigating complex anatomical pathways and detecting subtle tissue properties. Current research into whisker-based sensing is primarily focused on understanding the underlying neural mechanisms, circuits, and processing pathways that govern whisker functionality [33,34]. Additionally, theoretical mathematical models are being explored to translate these biological principles into practical applications. Such biomimetic approaches have led to innovative solutions across various domains, including obstacle avoidance in robotics and autonomous vehicles [35], terrain-based navigation enhancement [36], vortex monitoring and flow sensing in unmanned underwater vehicles [37], and precise tasks such as contact force measurement, airflow detection, and surface roughness discrimination [38–40].

Inspired by this sensory mechanism, we present a multichannel whisker system as an add-on tactile sensing modality for endoluminal intervention. Designed to complement rather than replace vision-based endoscopy, the present proof-of-concept study focuses on 3 clinically relevant tactile primitives that are difficult to access through vision alone, namely, surface texture discrimination, local geometric perception at millimeter scale, and early radial contact sensing during confined navigation. These targets define the present proof-of-concept scope and reflect clinically relevant tactile sensing objectives for subtle mucosal irregularity, local geometric deviation, and instrument–tissue interaction in endoluminal use. To assess these capabilities in a controlled and interpretable approach, we combine benchmark and phantom-based experiments that test whether a common biomimetic sensing platform, together with modular sensor mounts, shared acquisition, and calibration architecture, can support multiple tactile readouts relevant to endoluminal intervention across different task configurations.

The contributions of this work are as follows. First, we design and implement a compact multichannel whisker hardware system that integrates dedicated signal-conditioning circuitry with a high signal-to-noise ratio (SNR), modular sensor mounts, and synchronous tactile acquisition, enabling dynamic and quasi-static sensing for add-on use in endoluminal intervention. Second, we establish an affine transformation-based calibration and decoding framework that improves cross-channel consistency and measurement reliability by compensating for manufacturing imperfections and installation variability, thereby supporting interpretable downstream tasks such as geometric reconstruction, and radial force estimation. Third, we provide proof-of-concept validation of the system in both robotic and handheld settings through representative experiments in texture discrimination, local shape reconstruction, and radial force sensing with collision-aware interaction monitoring. These results establish a practical baseline for future development of whisker-based tactile sensing to augment vision-centric endoluminal workflows. Future work will further refine the hardware design, extend validation in clinically representative settings, and explore learning-based multimodal modelling to strengthen inference robustness and evaluate the role of whisker-based tactile sensing in endoscopic screening and robotic assistance.

## Materials and Methods

### Conceptual design and modeling analysis

**Fig. 1.**
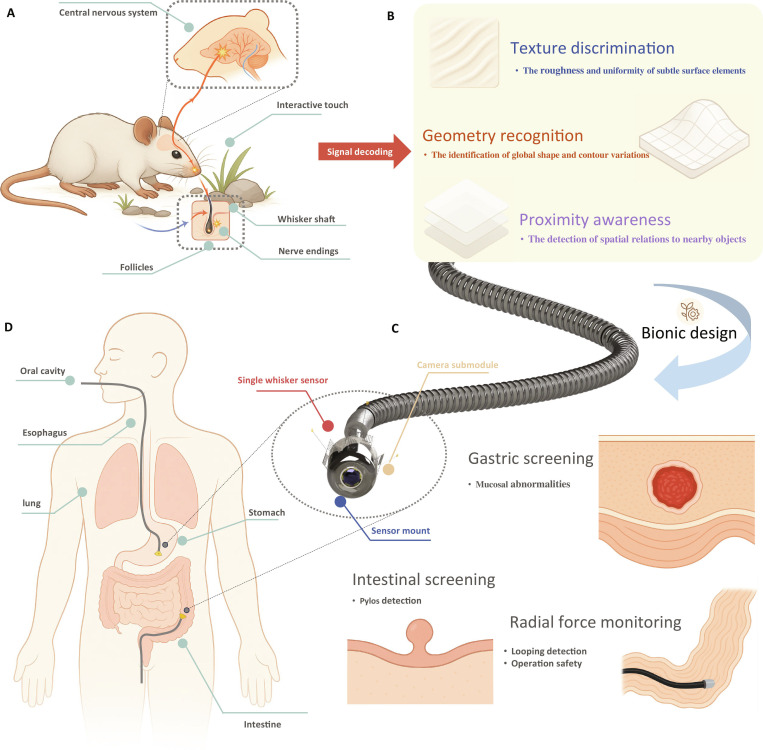
Conceptual design of the multichannel whisker add-on for endoluminal screening. (A and B) Biological basis: rodents engage in active whisking, with tactile signals decoded by the central nervous system to infer surface texture, geometry, and spatial proximity, enabling navigation and object recognition in constrained environments. (C) Engineering implementation: a concentric array of flexible, instrumented whiskers is integrated at the distal tip, transducing deflections into spatially encoded electrical signals that reflect contact dynamics along the examination path. (D) Clinical integration: during gastrointestinal screening, these signals are processed through algorithmic pipelines to extract local mechanical properties of mucosal tissues, thereby supporting lesion identification and improving diagnostic accuracy.

Fig. 1 illustrates the conceptual design of a multichannel whisker system designed for clinical applications. In biological contexts, rodents utilize tactile information from dynamic whisker–environment interactions, leveraging on the sophisticated neural decoding capabilities of their central nervous systems to interpret spatial and structural characteristics of their surroundings (Fig. 1A and B). This biological strategy facilitates navigation and perception within constrained environments. Inspired by this biological paradigm, the proposed hardware system acquires tactile information through direct interaction with tissue during clinical screening (Fig. 1C and D). The resultant tactile signals encode crucial pathological characteristics of the inspected mucosal tissues. Subsequently, the system decodes these signals, translating them into clinically relevant information.

To effectively realize this concept, a precise biomimetic system with robust tactile sensing and computational methods for signal conditioning and inference is essential. Comprehensive modeling and analysis of the whisker sensing mechanism are therefore indispensable, providing critical insights to optimize both the design and practical deployment of the proposed system. Recent advancements in Refs. [41–44] have demonstrated substantial progress in whisker modeling methodologies, predominantly employing cantilever beam theory. These modeling frameworks, encompassing both static and dynamic approaches, shown in Fig. S1, utilize distinct boundary conditions to accurately characterize specific mechanical behaviors. Under elastic beam theory, the mechanical behavior of whisker-like structures is mathematically formulated as Eq. (1):

$$\mathrm{EI}y^{\prime \prime \prime }=-\mathrm{Pu}\left(x-d\right)+C_{1}$$ (1)

*E*, *I*, and *y* denote Young’s modulus, momentum of inertia, and deflection of the whisker, respectively. *P*, *x*, *d*, and *C*_1_ represent the force applied, observation point, distance to be measured, and coefficient to be determined, respectively. The step function is denoted by *u*. In static scenarios, the deployment of multiple sensors $\left(x_{1},\;\theta_{1}\right)$ and $\left(x_{2},\;\theta_{2}\right)$ along the beam enables the estimation of the distance from the detected object to the fixed point on the beam.

$$d=\frac{\frac{\tan\theta_{2}}{\tan\theta_{1}}x_{1}^{2}-x_{2}^{2}}{2\left(\frac{\tan\theta_{2}}{\tan\theta_{1}}x_{1}-x_{2}\right)}$$ (2)

In dynamic scenarios, leveraging external actuators such as motors and encoders facilitates a more concise estimation of this information.

$$d=\frac{3\mathrm{EI}\tan\beta \;}{\tau }\approx \frac{3\mathrm{EI}\;\beta }{\tau }$$ (3)

In this study, the dynamic model, based on simplified sensor deployment, is adopted in the following design due to its improved robustness, accuracy, and fast screening capability [43] relative to other approaches.

### Design and implementation

The proposed whisker-based hardware solution encompasses specific components, including the whisker sensor and sensor mount design, the signal conditioning circuit design, and the corresponding data decoding algorithm design.

#### Whisker sensor and mount designs

The whisker sensor itself is positioned at the forefront of the proposed solution. Its primary function is to transduce tactile interaction information between the whisker and the probed target into electrical signals. These signals are subsequently captured by downstream signal conditioning circuits, thereby facilitating the extraction of information such as tissue texture, stiffness, and structural details. A typical whisker sensor comprises the whisker itself and a transducer located at the base of the whisker, emulating the function of a follicle. Whisker designs exhibit considerable variability, ranging from those replicating natural animal whiskers to metallic strings and specially prepared composite materials, among others. Variances in diameter, structure, elasticity, and mass distribution directly influence the natural frequency of the whisker sensor, thereby impacting the captured signals, which will be discussed in Discussion. In this study, 0.16-mm acupuncture needles were used as whisker shafts, with their slender metallic geometry providing a practical balance between compliance and dynamic responsiveness for the present prototype, enabling measurable deflection under light contact while preserving sensitivity to transient tactile fluctuations during sweep-based sensing. Each distal tip was shaped and covered with a polyimide film to soften the terminal contact region and provide partial protection during wall interaction.

The design of the transducer exhibits the most direct and critical influence on signal conversion. Prior research has extensively focused on designing and implementing various transducer schemes to simulate real follicles effectively, with the goal of achieving optimal outcomes in specific application scenarios. Historical and contemporary approaches encompass capacitive microphones, strain gauges, capacitor plates, laser diodes, Hall effect sensors, triboelectric nanogenerators, pressure sensors, and others. Each scheme involves its distinct merits and drawbacks, necessitating careful consideration and trade-offs among various factors, that depend on the specific application context. In our prior investigations, a polyvinylidene fluoride (PVDF)-based approach [45] was employed to achieve robust dynamic sensing capabilities while maintaining a simplified preprocessing circuit design. However, because of the inherent characteristics of piezoelectric effects, this approach is incapable of measuring quasi static responses. In this study, we adopt a strain gauge-based approach, as introduced in Ref. [46], showcasing high sensitivity and preserving both dynamic and quasi static sensing capabilities. Moreover, an optimized preprocessing circuit design based on integrated chips effectively reduces system volume, emphasizing this method as a potential and advantageous alternative to the PVDF-based solution.

**Fig. 2.**
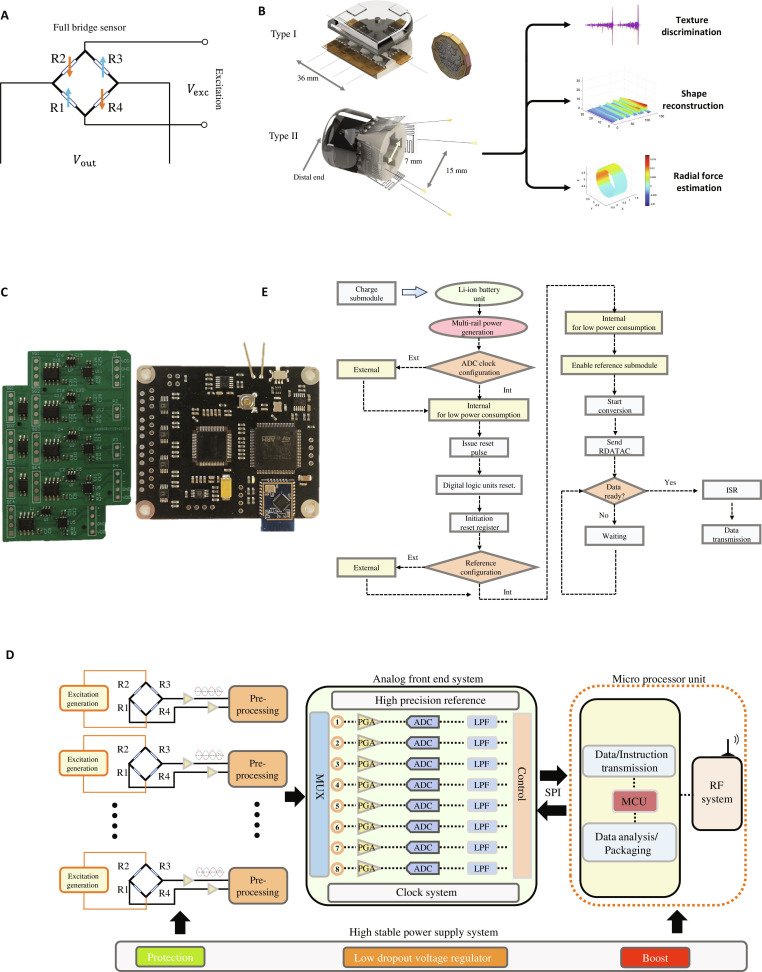
System overview of the proposed multichannel whisker system, illustrating (A) the whisker sensor design, (B) mounting architecture, (C) hardware prototype, (D) hardware framework, and (E) embedded algorithm pipeline is provided in Fig. S4. The system enables synchronous multichannel tactile signal acquisition and supports functions such as texture discrimination, shape reconstruction, and radial force estimation to complement vision-based endoluminal inspection.

Perceiving tactile interaction through whiskers as a probing challenge emphasizes the significance of a multichannel whisker system in augmenting detection special resolution and screening efficiency. In alignment with our proposed synchronous multichannel signal conditioning system with 8 channels, we have developed 2 distinctive whisker sensor mounts to arrange the spatial distribution of multiple whiskers. As illustrated in Fig. 2B, the sensor mount Type I consists of 2 components, an upper and a lower part, each measuring 36 mm × 36 mm × 8 mm. Four whiskers are positioned on the underside to capture fine details such as texture and structure, while additional whiskers arranged around the periphery provide radial sensing to detect proximity and contact from multiple directions. Furthermore, sensor mount Type II, depicted in Fig. 2B, was developed as an initial prototype for radial force estimation and collision detection in a procedurally relevant endoluminal setting. The current mount has an outer diameter of 15 mm and a central through-hole of 7 mm, intended to preserve a potential working channel in the prototype design. In this configuration, each whisker shaft is angled at 20° relative to the sensor mount axis, with a shaft length of 40 mm, providing the capability to perform radial force estimation and collision detection. Although the present form factor remains larger than desirable for routine integration with standard GI endoscopes, it supports the evaluation of tactile sensing for safer and more controlled endoscopic navigation and provides a basis for future miniaturization. This task-specific differentiation further points to a future workflow in which navigation-oriented configurations support radial contact monitoring during advancement, whereas structure-sensitive configurations enable controlled short-range interrogation of visually ambiguous local regions.

#### Signal conditioning circuit design

The whisker sensor converts tactile signals into electrical measurements, requiring additional signal processing for subsequent transmission and analysis.

##### Preprocessing module

Typically, the high sensitivity of a strain gauge constitutes its primary advantage, yet paradoxically rendering it susceptible to interference. The strain gauge translates signals such as vibration and deformation into variations in resistance, with 2 main categories including metal foil and semiconductor types. In this study, we select the former, wherein the resistance fluctuation of this strain gauge type primarily arises from the axial strain-induced length change in the metal foil. Its static resistance is defined by Eq. (4), wherein *L*, *S*, *r*, and *ρ* denote the sensitive grid length, cross-sectional area, radius, and resistivity of the strain gauge, respectively.

$$R=\rho \cdot \frac{L}{S}=\rho \cdot \frac{L}{\pi r^{2}}$$ (4)

A common limitation of the high sensitivity of strain gauges is their susceptibility to temperature fluctuations, which introduces unreliability into strain measurements and also introduces issues of linearity degradation. In our preprocessing module, we introduce a Wheatstone bridge structure to address the aforementioned challenges, as depicted in Fig. 2A. Generally, the static resistance of the bridge components is configured as *R*1 = *R*3 = *R*2 = *R*4. When subjected to external stress,

$$\begin{array}{c} V_{\text{out}}=\left(\frac{R1+∆R1}{R1+∆R1+R2+∆R2}-\frac{R4+∆R4}{R3+∆R3+R4+∆R4}\right)*\\ V_{\mathrm{exc}}\approx \frac{R1R2}{{\left(R1+R2\right)}^{2}}\left(\frac{∆R1}{R1}-\frac{∆R2}{R2}+\frac{∆R3}{R3}-\frac{∆R4}{R4}\right)*\\ V_{\mathrm{exc}}=\frac{V_{\mathrm{exc}}}{4}\left(\frac{∆R1}{R1}-\frac{∆R2}{R2}+\frac{∆R3}{R3}-\frac{∆R4}{R4}\right)\\ \;=\frac{V_{\mathrm{exc}}}{4}K_{0}\left(\varepsilon_{1}-\varepsilon_{2}+\varepsilon_{3}-\varepsilon_{4}\right) \end{array}$$ (5)

where $K_{0}$ denotes the gauge factor of the strain gauge, and $V_{\mathrm{exc}}$ is the reference voltage. From Eq. (5), it is evident that the Wheatstone bridge achieves a state of balance, resulting in an output of zero when not subjected to force. Throughout the application process, errors induced by temperature fluctuations are effectively mitigated. Moreover, a dedicated precision voltage reference serves as the excitation source to drive the Wheatstone bridge, ensuring high stability of the signal source. A single-stage programmable differential amplifier is then employed to provide signal preamplification, concurrently achieving bidirectional signal perception under a unipolar power supply.

The design details above describe the single-channel implementation. Considering the requirement for independence among multiple channels, the preprocessing modules are organized into 2 independent groups, each consisting of 4 channels, forming a complete 8-channel preprocessing system. Fig. 2C illustrates the prototype of the preprocessing module, with each module having dimensions of 33.8 mm × 51.0 mm × 1.8 mm, ensuring efficient integration and scalability within the overall system architecture.

##### Analog front end module

The preprocessing module transforms the tactile output from the whisker sensor into a subtle differential voltage signal. Nonetheless, this signal persists in analog form and requires quantization into digital values for subsequent analysis. To ensure precise timing consistency in multichannel whisker signals, a multichannel Σ-Δ analog front-end architecture is employed as the signal conditioning framework. In contrast to conventional designs relying on discrete components, this architecture incorporates low-noise operational amplifiers, low-power high-precision bandgap references, temperature compensation, noise shaping, oversampling, and other techniques. This approach substantially enhances system integration, minimizes system size, improves SNR, reduces inherent noise, and guarantees precise channel-to-channel matching and timing control.

As illustrated in Fig. 2D, the raw analog voltage signal output from the preprocessing module is multiplexed and directed to independent signal processing channels. Within these channels, the signal undergoes digital programmable amplification, analog-to-digital conversion, and filtering processes, ultimately converted into a low-noise digital representation of the original signal. Throughout this sequential process, assuming a unity gain setting, the resolution of the 24-bit high-precision analog-to-digital converter (ADC) can be defined by Eq. (6). These signal processing procedures are executed through the Serial Peripheral Interface communication protocol, under the control of a microprocessor unit, and involve interactive data and command exchanges with the analog front-end module. This ensures the robustness of digital logic and signal flows.

$$V_{\mathrm{LSB}}=\frac{V_{\mathrm{Ref}}}{2^{23}}\approx 0.5\;\mu \text{V}$$ (6)

##### Microprocessor unit and power supple module

As previously discussed, the microprocessor unit, acting as the central control unit, not only coordinates various components such as AFE but also performs signal processing tasks while managing the overall behavior of the system. In the proposed whisker system, the captured digitalized tactile information is wirelessly transmitted to terminal devices for signal analysis and parsing. We also retain a wired communication option to mitigate potential signal attenuation in future miniaturized implementations of the propose system intended for in-body deployment. A wireless data transmission protocol has been designed to facilitate high-speed and secure data transfer. According to this protocol, the microprocessor unit, based on the STM32 architecture, packages the acquired data into signal frames. These frames undergo bidirectional signal transmission with the RF module by initializing modulators, thereby completing the wireless transmission of data. A cyclic redundancy check (CRC) is incorporated into this process for error detection.

The Power Supply module generates multiple power rails to accurately supply power to all analog and digital units. Through circuit topology planning and optimized component layout, a well-isolated analog-to-digital unit is guaranteed, thereby maximizing the reduction of inherent system noise, minimizing signal reflection, and enhancing system stability and robustness.

#### Data decoding algorithms

While our previous work [45] explored end-to-end deep learning approaches for analyzing whisker-based tactile signals, enabling direct inference of outcomes such as disease recognition, this study adopts a foundational approach to ensure data consistency and reliability across channels before engaging higher-level analysis in the further. End-to-end processing is effective; however, retaining the capacity for direct interpretation of the whisker system scanning results is crucial for applications requiring immediate and reliable tactile feedback.

The calibration process, inspired by a rat’s natural ability to adapt to structural changes or damages, is used to achieving this consistency. In the natural world, rats continue to accurately perceive their environment even with damaged whiskers, as their sensory systems recalibrate to maintain functional resilience. This adaptability highlights the importance of a resilient calibration system in our proposed artificial whisker system, as even minor deviations in sensor output introduced by manufacturing and installation variances or imperfections across channels can substantially impact data reliability. To replicate this adaptability, we first modeled the signal propagation chain, addressing noise sources and their cumulative impact on signal fidelity across stages. This approach allows us to correct for discrepancies in gain and mitigate noise that could compromise the sensor’s output quality.

As shown in the framework in Fig. 2D, assume that $S_{i}\left(t\right)$ represents the true tactile measurement captured by each whisker sensor, containing raw signal encoding the interaction information. The input signal for channel i can be expressed as:

$$V_{\text{in},i}\left(t\right)=S_{i}\left(t\right)+n_{\text{sensor},i}\left(t\right)$$ (7)

where $n_{\text{sensor},i}\left(t\right)$ represents the noise from the sensor, such as thermal noise and initial drift. After feeding the signal $V_{\text{in},i}\left(t\right)$ into the first preprocessing stage, the signal is amplified, introducing gain and noise specific to this stage. This yields the output as Eq. (8):

$$V_{\text{prepro},i}\left(t\right)=G_{1i}\left[V_{\text{in},i}\left(t\right)+n_{\text{preproc},i}\left(t\right)\right]$$ (8)

where $\;G_{1i}=G_{1}+∆G_{1i}$ is the actual gain of the preprocessing stage, and $n_{\text{preproc},i}\left(t\right)$ refers to input-referred noise of the preprocessing stage. The preamplified signal $V_{\text{prepro},i}\left(t\right)$ will be fed into the analog front-end system subsequently to complete filtering and digitalizing processes, resulting in the addition of further gain and noise inevitably, represented as:

$$V_{\text{out},i}\left(t\right)=G_{2i}\left[V_{\text{prepro},i}\left(t\right)+n_{\mathrm{ADC},i}\left(t\right)\right]$$ (9)

Similarly, $G_{2i}=G_{2}+∆G_{2i}$ is the actual gain of the AFE stage, and $n_{\mathrm{ADC},i}\left(t\right)$ is the noise introduced by the ADC.

Let $G_{\mathrm{tot},i}=G_{1i}G_{2i}$, the output of the final stage can thus be expressed as:

$$\begin{array}{c} \begin{array}{c} \begin{array}{c} \;V_{\text{out},i}\left(t\right)=\\ \underset{\text{The measurment signal}}{\underbrace{G_{\mathrm{tot},i}S_{i}\left(t\right)}}+\underset{\text{The noise contribution}}{\underbrace{G_{\mathrm{tot},i}n_{\text{sensor},i}\left(t\right)+G_{\mathrm{tot},i}n_{\text{preproc},i}\left(t\right)+G_{2i}n_{\mathrm{ADC},i}\left(t\right)}} \end{array} \end{array} \end{array}$$ (10)

As per Eq. (10), the output of the final stage consists of the measurement signal and the noise contribution item. The source of the error can be identified as gain discrepancies, resulting in each channel having a slightly different total gain $G_{\mathrm{tot},i}$, leading to interchannel inconsistencies and nonlinear response in the output signals, and accumulated noise contributions, degrading the signal quality, affecting measurement accuracy, and introducing the bias.

In semiconductor design, various types of noise contribute to fluctuations in signal integrity, each with distinct origins and characteristics. Common noise types include thermal noise (also known as Johnson–Nyquist noise), which arises from the random thermal motion of charge carriers; shot noise, resulting from the discrete nature of charge carriers crossing potential barriers; and flicker noise (or 1/f noise), typically caused by material defects or variations in carrier mobility. Within a given temperature range and target frequency band, and considering factors such as device aging over its expected lifespan, thermal and shot noise can be treated as random processes with constant power spectral density (PSD). Despite introducing unpredictable fluctuations in the signal over time, these noise sources maintain a relatively stable PSD, making it feasible to apply calibration techniques to compensate for gain discrepancies across channels and minimize the influence of noise. Herein, we proposed the affine transformation method to achieve this.

An affine transformation is a linear mapping method that preserves points, straight lines, and planes, involving scaling, translation, and rotation. In our multichannel whisker calibration, assume that we have ***N*** whisker sensors, N=1,2,…,N, for each sensor i, we could define an affine transformation as:

$$y_{\mathrm{ref}}=X_{i}\beta_{i}+\epsilon_{i}$$ (11)

where $X_{i}$ is a T×2 matrix as shown in Eq. (12), in which $x_{i}={\left[x_{i1},\;x_{i2},\ldots ,\;x_{\mathrm{iT}}\right]}^{T}$ is the raw measurement from the ith whisker sensor. T is the sampling time stamp, and $y_{\mathrm{ref}i}$ is the ground-truth data corresponding to the sensor i.

$$X_{i}=\left[\begin{array}{cc} x_{i1} & 1\\ x_{i2} & 1\\ \vdots & \vdots \\ x_{\mathrm{iT}} & 1 \end{array}\right]$$ (12)

Meanwhile, $\beta_{i}$ and $\epsilon_{i}$ are the parameter vector and the residual vector, respectively, and defined as:

$$\beta_{i}=\left[\begin{array}{c} a_{i}\\ b_{i} \end{array}\right]\;\epsilon_{i}=\left[\begin{array}{c} \epsilon_{i1}\\ \epsilon_{i2}\\ \vdots \\ \epsilon_{\mathrm{iT}} \end{array}\right]$$ (13)

Extending the fitting process to all ***N*** sensors by stacking the data, we could calibrate all the whisker sensors so that the interchannel variability could be improved.

$$\begin{array}{c} \begin{array}{c} \begin{array}{c} \left[\begin{array}{c} y_{\mathrm{ref}1,1}\\ \begin{array}{c} y_{\mathrm{ref}1,2}\\ \vdots \\ \begin{array}{c} \begin{array}{c} y_{\mathrm{ref}1,T}\\ \begin{array}{c} y_{} \end{array}\\ y_{\mathrm{ref}2,T}\\ \vdots \end{array} \end{array} \end{array}\\ y_{\mathrm{ref}N,1}\\ y_{\mathrm{ref}N,2}\\ \vdots \\ y_{\mathrm{ref}N,T} \end{array}\right]={\left[\begin{array}{ccccccc} x_{11} & 1 & 0 & 0 & \cdots & 0 & 0\\ x_{12} & 1 & 0 & 0 & \cdots & 0 & 0\\ \vdots & \vdots & \vdots & \vdots & \ddots & \vdots & \vdots \\ x_{1T} & 1 & 0 & 0 & \cdots & 0 & 0\\ 0 & 0 & x_{21} & 1 & \cdots & 0 & 0\\ 0 & 0 & x_{22} & 1 & \cdots & 0 & 0\\ \vdots & \vdots & \vdots & \vdots & \cdots & \vdots & \vdots \\ 0 & 0 & x_{2T} & 1 & \cdots & 0 & 0\\ 0 & 0 & 0 & 0 & \ddots & 0 & 0\\ 0 & 0 & 0 & 0 & \cdots & x_{N1} & 1\\ 0 & 0 & 0 & 0 & \cdots & x_{N2} & 1\\ \vdots & \vdots & \vdots & \vdots & \cdots & \vdots & \vdots \\ 0 & 0 & 0 & 0 & \cdots & x_{\mathrm{NT}} & 1 \end{array}\right]}_{\mathrm{NT}\times 2N}\left[\begin{array}{c} a_{1}\\ b_{1}\\ a_{2}\\ b_{2}\\ \vdots \\ a_{N}\\ b_{N} \end{array}\right]+\left[\begin{array}{c} \epsilon_{11}\\ \epsilon_{12}\\ \vdots \\ \epsilon_{1T}\\ \epsilon_{21}\\ \epsilon_{22}\\ \vdots \\ \epsilon_{2T}\\ \vdots \\ \epsilon_{N1}\\ \epsilon_{N2}\\ \vdots \\ \epsilon_{\mathrm{NT}} \end{array}\right] \end{array} \end{array} \end{array}$$ (14)

Generally, retrieving the parameter vector $\beta =\left[a_{1},b_{1},a_{2},\right.$ ${\left.\;b_{2},\ldots ,\;a_{N},\;b_{N}\right]}^{T}$ can be obtained by adopting the ordinary least squares method as:

$$\hat{\beta }={\left(X^{T}X\right)}^{-1}\;X^{T}\;y_{\mathrm{ref}}$$ (15)

However, in practical scenarios, measurement noise, sensor faults, or environmental factors can all introduce outliers during the parameter calculation stage, degrading calibration performance of the fitting model. To mitigate this, herein, the bisquare (also known as Tukey’s bisquare) weight function, as one of the robust fitting methods, is used for this purpose, which assigns weights to each data point based on the residuals and is defined as:

wr=1−rc22,r<c0,r≥c (16)

where r is the standardized residual, and c is tunning constant, normally set as 4.685, which determines the cutoff point beyond which residuals are considered as outliers and assigned to zero weight. By weighting residuals appropriately, the bisquare function improves the calibration model’s resilience to outliers, yielding more stable and reliable parameter estimates. Therefore, the objective function for this nonlinear regression problem to retrieve the parameter vector $\hat{\beta }\;$ can be redefined as:

$$\min_{a_{i},b_{i}}\sum_{t=1}^{T}w_{t}{\left(y_{\mathrm{ref}i,t}-\left(a_{i}x_{\mathrm{it}}+b_{i}\right)\right)}^{2}$$ (17)

Overall, this calibration framework is intended to improve cross-channel consistency and measurement interpretability in the multichannel whisker system, thereby supporting downstream tasks such as shape reconstruction, radial force estimation, and collision detection.

In the present system, the affine transformation is primarily intended to compensate for channel-wise gain and offset mismatch arising from manufacturing tolerances, mounting variability, and accumulated analog front-end discrepancies. This linear calibration is well suited to the dominant error structure observed in the current hardware, where interchannel inconsistency is mainly expressed as differences in sensitivity and baseline level under a fixed experimental configuration. At the same time, the method does not explicitly model stronger nonlinear effects, such as contact-condition-dependent hysteresis, posture-dependent coupling, or time-varying drift beyond the calibration condition. Accordingly, the present approach should be interpreted as a practical calibration strategy that improves cross-channel consistency and interpretability under the current experimental conditions, while more expressive calibration models may become beneficial as future implementations move toward broader operating conditions and more complex sources of interchannel nonlinearity.

#### Experimental setup

To evaluate the performance of the multichannel whisker system, we employed a dual-mode experimental setup encompassing both controlled actuation mode and manual actuation modes: a robotic actuation system for high-precision trajectory execution and a manual operation mode to capture the subtleties of human-guided interactions.

**Fig. 3.**
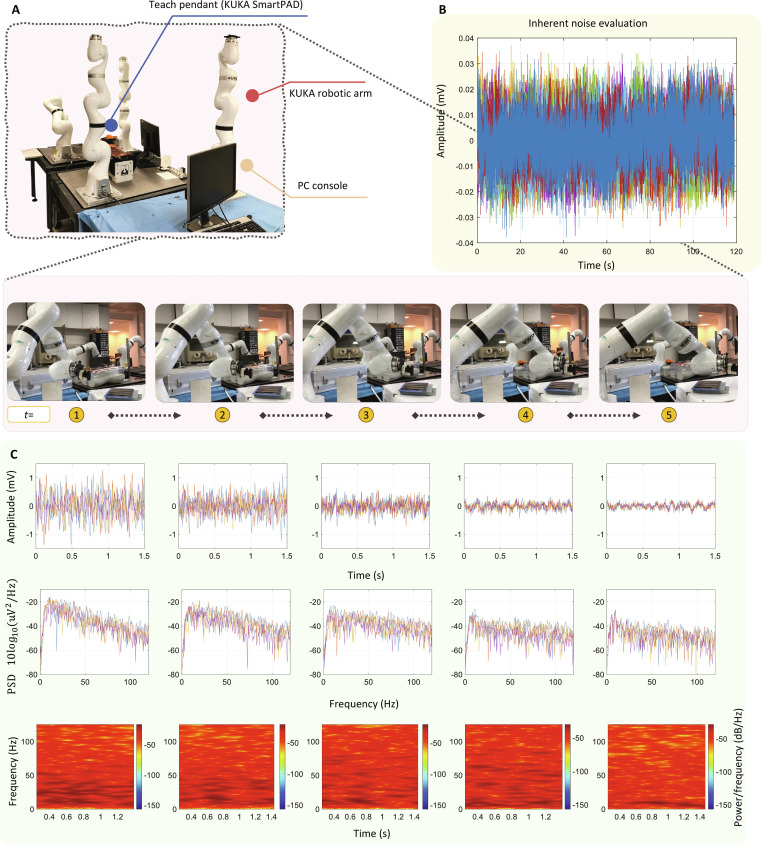
Benchmarking experiments illustrating the controlled actuation setup, inherent noise characterization, and texture discrimination analysis for performance evaluation. (A) Robotic actuation platform for controlled evaluation of the multichannel whisker hardware system. The whisker add-on was mounted at the end effector, enabling reproducible contact kinematics for calibration and benchmarking studies. (B) Inherent noise evaluation of the multichannel whisker hardware system. (C) Experimental results of texture discrimination using sandpapers of varying grit sizes (P80 to P2000). The top row shows the 5 tested samples. The second, third, and fourth rows present the corresponding whisker outputs in the time domain, frequency domain via power spectral density, and time–frequency domain, respectively.

In the controlled trials, a robotic arm (LBR iiwa 7 R800, KUKA Robotics Corp), shown in Fig. 3A, was employed due to its precise *XYZ*-axis control capabilities, offering a positional repeatability of ±0.1 mm and a maximum reach of 800 mm. The whisker system was mounted on the end effector of the robotic arm, allowing for the execution of predefined motion trajectories through centralized controller input, thus ensuring consistency and repeatability across trials. Complementing this, manual operation of the whisker system was incorporated to capture the detailed dynamics and variability of human-driven interactions, discussed further in the respective experimental sections separately. This combined approach provided a comprehensive assessment of the system performance under automated conditions, as well as its adaptability in user-controlled scenarios.

## Results

To evaluate the proposed system, we combined system characterization with controlled benchmark experiments and a phantom-based pilot study. The evaluation was organized around 3 tactile primitives relevant to future endoluminal use, namely, texture discrimination, local geometric perception at the millimeter scale, and early radial contact sensing. Within this framework, the benchmark experiments isolate texture and local geometric sensing, whereas the phantom-based study assesses the procedural relevance of early radial contact sensing in an endoluminal setting.

### System characterization and inherent noise evaluation

A high SNR is essential for converting subtle, externally induced deformations into measurable electrical signals, which serve as the foundation for extracting and analyzing tactile modality features of interest. In our signal chain propagation model, represented by Eq. (10), the output signal $\;V_{\text{out},i}\left(t\right)$ comprises both the measurement signal and various noises. These noise components substantially impact the overall SNR of the whisker system and play a critical role in parameter selection within the affine transformation, which enables us to mitigate interchannel variability. Consequently, a quantitative evaluation of inherent noise levels is a prerequisite for realizing the full potential of the whisker modality, and providing baseline for iterative hardware refinement and offering an objective assessment of measurement stability and repeatability across trials.

In this section, we present an inherent noise evaluation, consisting of 5 experimental trials, each capturing over 30,000 consecutive conversion points sampled at 250 Hz. As shown in Fig. 3B, the results demonstrate a stable and consistent noise distribution across all 8 independent input channels at room temperature, with an RMS noise level of 0.41 μV and a peak-to-peak noise of 2.91 μV. Furthermore, throughout prolonged pressure testing, the system temperature rise, attributed to dissipated heat, remains less than 2 °C, ensuring thermal safety for prospective in vivo applications.

The noise evaluation experiments indicate that the inherent system noise level is substantially lower than the whisker response signal observed under typical interaction processes. This low inherent noise not only minimizes the need for extensive postprocessing noise reduction but also provides a robust foundation for accurately extracting features of interest. The exceptional SNR achieved enhances the system sensitivity and reliability, ensuring that even subtle textural or structural variations can be detected with high precision, which is critical for advanced applications.

### Evaluation of tactile primitives

#### Texture discrimination benchmark

In this section, we evaluate texture discrimination as one tactile primitive of the proposed system, motivated by the requirement to capture subtle surface irregularity in endoluminal sensing. Texture is represented here by fine-scale differences in surface roughness and uniformity. Tactile data were acquired through an interactive scanning sequence executed by the KUKA robotic arm. Whereas pronounced texture differences were distinguished in our previous work [45], the present experiment was designed to assess performance under more challenging conditions. To this end, 5 grades of sandpaper, ranging from P80 to P2000, were selected as repeatable roughness benchmarks for systematic tactile acquisition.

The standard interactive trial with each sample consists of 4 sequential stages. Initially, the proposed whisker system remains static, producing a baseline output in the time-domain signal, where the calibrated baseline across channels consistently maintains a near-zero value. As the system begins moving, minor jitter signals appear, superimposed on the baseline output, resulting from vibrations induced by the robotic actuator. Once the whisker shaft contacts the test surface, the signal transitions to a rising phase, reaching a peak as the whisker fully engages the surface. In the subsequent sweeping stage, the system encodes texture information from the target surface into the output signal. The final stage occurs as the whisker disengages, releasing stored elastic potential energy and generating an oscillatory signal as it retracts.

Fig. 3C presents the results of the interactive trials extracted from the sweeping stage, shown in the time domain, frequency domain, and time–frequency domain. Before analysis, each sweep segment was baseline corrected by removing a dominant constant offset from the precontact period and then detrended using locally estimated scatterplot smoothing (LOESS) to suppress low-frequency drift while preserving the texture-related dynamic component. For enhanced detail discrimination, we utilized 4 whiskers positioned beneath the sensor mount (Type I) as independent sensing channels. In the time domain, signals from these 4 channels consistently reflect clear distinctions in response to varying surface roughness. Coarser textures, such as P80, produce higher-amplitude fluctuations, attributed to the stronger interaction forces and larger surface features encountered by the whisker tips. Conversely, finer textures like P2000 yield more stable, lower-amplitude signals, indicating weaker, more uniform interactions. This demonstrates the system ability to capture textural variations effectively and highlights its sensitivity to tactile fine-grained variations.

The frequency domain analysis further supports these observations. Coarse textures are dominated by low-frequency components due to the larger surface features, which induce slower, more distinct response patterns. As the texture becomes finer, frequency components shift to higher ranges, capturing the finer, more rapid interactions characteristic of smoother surfaces. This spectral shift underscores the system capability to detect subtle changes in texture, even across closely spaced roughness levels, and validates the reliability of frequency-based feature for texture discrimination.

In the time–frequency domain, the analysis focuses on a single channel to highlight transient frequency shifts throughout the interaction. For coarser textures, spectrograms reveal concentrated energy in lower frequencies, reflecting sustained impacts of larger surface features. Finer textures, however, display dispersed energy in higher frequencies, aligning with the rapid, finer-scale interactions of the whisker tip with smoother surfaces. Despite representing only one channel due to the nature of time–frequency analysis, similar patterns across other channels indicate stable, reproducible cross-channel performance, further validating the whisker system ability to capture transient, time-varying frequency components with high precision.

It is important to note that the whisker system is not limited to distinguishing sandpaper textures alone. Sandpaper was selected not as a direct surrogate for colonic mucosa, but as a controlled roughness ladder for testing sensitivity to fine surface irregularity, a sensing dimension that is clinically relevant because subtle mucosal abnormalities are often defined by low-contrast textural and topographic change [18].

#### Shape reconstruction benchmark

Local geometric perception is another tactile primitive targeted in the present study. Unlike texture discrimination, which emphasizes the dynamic response during the sweeping stage, shape reconstruction depends on information distributed across the full interaction trial. Clinically, millimeter-scale changes in luminal geometry, including early narrowing, low-profile protrusions, and subtle postintervention topography, may be difficult to discern reliably under nonideal viewing conditions. Complementary tactile surface mapping is therefore relevant not only to local structural assessment, but also to navigation in confined endoluminal environments. In this context, the present experiment was designed to determine whether the proposed whisker system could capture controlled local height and contour variation with sufficient sensitivity to support relative shape reconstruction.

To provide a controlled geometric benchmark, we used 2 sets of 3-dimensional (3D)-printed test samples, each measuring 150 mm × 60 mm. Both sets incorporated wavy, rectangular, and triangular cross-sectional features with stepped elements in 1.5-mm increments. This increment was selected to assess geometric sensitivity at a clinically meaningful scale, as many colorectal abnormalities occur in the millimeter range and superficially elevated lesions may be distinguished from polypoid morphology by only a few millimeters in height [47,48]. Within this benchmark design, Set I, with uniform height distribution, served as the initial case for calibration and reconstruction. Set II introduced tilted bump elements to evaluate performance under nonuniform local geometry and to test the robustness of the calibration under a more demanding reconstruction condition.

During data acquisition, 4 whisker channels mounted beneath sensor mount Type I captured raw time-series signals as the system scanned the surface. These signals were then processed using a triangulation-based natural neighbor interpolation method to increase the density of reconstruction points to provide a refined and continuous representation of the original surface mappings for a more intuitive visualization.

**Fig. 4.**
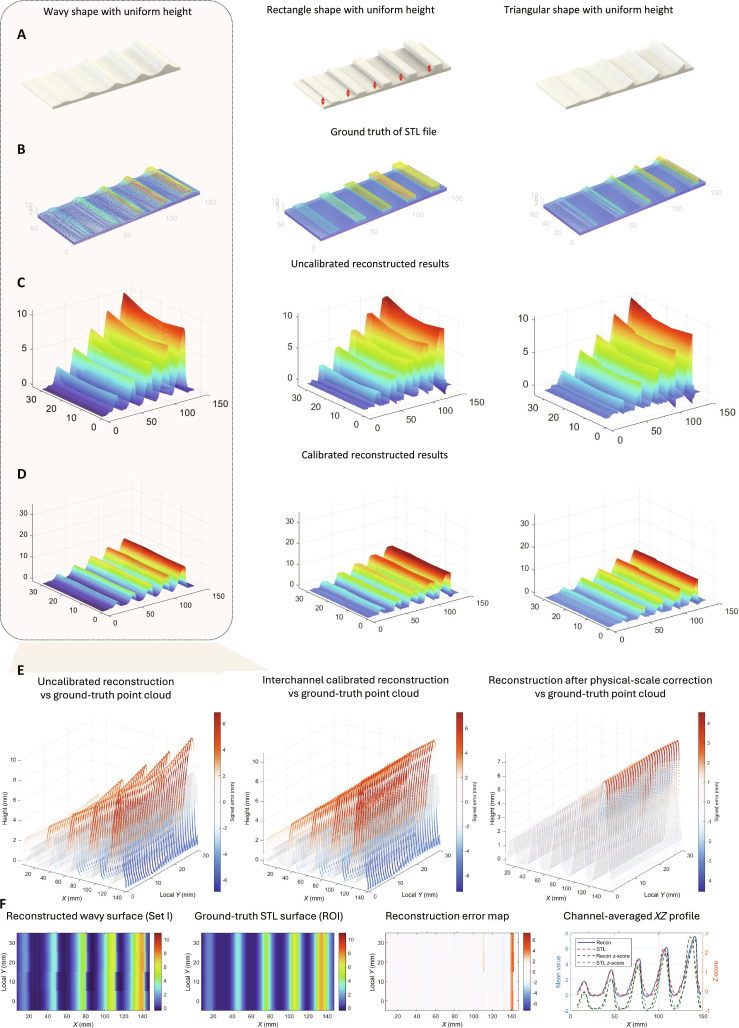
Shape-reconstruction benchmarking against STL-derived ground truth. (A) Benchmark objects with stepped geometric features. (B) STL-derived reference surfaces. (C) Uncalibrated reconstructed results. (D) Calibrated reconstructed results. (E) Point-cloud overlays comparing reconstructed surfaces with the STL-derived ground truth under uncalibrated, interchannel calibrated, and physical-scale-corrected conditions. (F) Representative quantitative comparison for the Set I wavy sample, including reconstructed and ground-truth 2D height maps, reconstruction error map, and the channel-averaged *XZ* profile with *z*-score-based topographic comparison.

In the uncalibrated results, there is an evident mismatch with the ground truth, which can be attributed to factors such as leveling issue in the installation plane of the test platform, multijoint calibration issues in the KUKA robotic arm, and minor imperfections in the manufacturing and installation of the whisker sensor array. These issues introduce nonlinear responses and interchannel variability, resulting in less accurate reconstructions when uncalibrated. To address these issues, we applied the affine transformation-based calibration as discussed before, which improved alignment with the ground-truth data by compensating for nonlinearities and ensuring consistency across channels. Calibration coefficients were determined from the first sample in Set I and then applied unchanged to the remaining benchmark objects to assess generalizability under a fixed correction. The effect of calibration was especially evident in Set II (fig. S6), where the whisker system more faithfully recovered the tilted bump elements, indicating that the affine transformation effectively compensated interchannel mismatch under nonuniform local geometry. To quantify reconstruction fidelity, we further compared the reconstructed local surface strip against the stereolithography (STL) file-derived ground-truth strip for all 6 benchmark objects using Pearson correlation, Spearman correlation, structural similarity index measure, cosine similarity, and *Z*-axis mean absolute error (MAE) (Table S2). A representative example is shown in Fig. 4F, which visualizes the reconstructed and ground-truth 2-dimensional height maps, the error map, and the channel-averaged *XZ* profile for the Set I wavy sample. Calibration produced a consistent improvement across the full benchmark set. The mean Pearson correlation increased from 0.081 to 0.796, the mean Spearman correlation increased from −0.055 to 0.630, and the mean *Z*-axis MAE decreased from 2.22 to 0.81 mm, corresponding to a 63.4% reduction. This improvement was observed for all 6 objects, including the more challenging tilted Set II geometries. For example, Pearson correlation increased from 0.278 to 0.930 for the Set I wavy sample and from −0.137 to 0.892 for the Set II tilted wavy sample, while the final postcalibration *Z*-axis MAE ranged from 0.53 to 1.27 mm across the benchmark set. Together, these results support millimeter-scale local geometric sensing within the present proof-of-concept configuration. Importantly, the calibration coefficients were channel-specific and remained stable across repeated experiments within the present hardware configuration, provided that sensor degradation and mounting changes were negligible. Under these conditions, the same coefficients could be reused in subsequent experiments, improving repeatability and robustness. By contrast, substantial changes in sensor replacement, mounting geometry, or acquisition conditions could alter the channel response and would require recalibration.

Regarding the spatial axes of the reconstruction, there are current limitations to the dimensional fidelity. The *X*-axis in the reconstructed images is derived from estimates based on the movement speed of the KUKA arm, as the whisker sensor lacks a mechanism of direct positional tracking. The *Y*-axis reflects the physical distribution of the 4 whisker channels, spaced at 9-mm intervals, covering a total span of 27 mm, which limits the scan coverage to approximately half of the 60-mm-wide test samples. This constrained span provides a partial mapping, capturing only the central portion of the test surface. For the *Z*-axis, calibration improves reconstruction fidelity under the present configuration, but the recovered height mapping remains dependent on the initial setup and mounting condition. In this study, our focus is on providing relative geometric mapping within a constrained area, aiming to enhance visualization capabilities for texture and shape in challenging environments, where vision-based methods may be limited due to nonideal lighting conditions. Future enhancements to the system will integrate additional sensors, such as inertial measurement units, to provide full spatial and kinematic data for more complete and absolute shape reconstructions.

Overall, the results underscore the whisker system high sensitivity and resolution in detecting surface geometry, even with complex profiles like the tilted elements in Set II. The calibrated reconstructions accurately reflect the distinctive cross-sectional features of each shape, including wavy, rectangular, and triangular, and capture subtle height variations introduced by the stepped bump elements. In the case of the tilted elements in Set II, the calibrated whisker system successfully captures the incline, indicating that the system can detect not only shape features but also variations in surface orientation. It is also noteworthy that the effective application of calibration through affine transformation not only improves cross-channel consistency but also markedly reduces the influence of external calibration errors, such as platform leveling and robotic alignment inaccuracies, and reinforces the system reliability and applicability in high-precision tactile sensing. This calibration procedure thus forms a foundational step toward achieving high-precision reconstructions, providing a robust framework for future system developments.

#### Phantom-based pilot study of radial force sensing

Following the benchmark experiments of texture discrimination and shape reconstruction, this study explores the application potentials of the whisker system in clinically relevant scenarios, focusing on radial force estimation with a complementary focus on collision detection. Radial force sensing provides essential tactile feedback in endoscopic procedures, where detecting subtle force variations is crucial for navigating confined anatomical structures and assessing tissue characteristics. To explore this potential, we conducted a phantom-based experiment simulating endoscopic screening, with the whisker system actuated in a handheld state to closely replicate real clinical conditions. This experiment underscores the system’s capacity to enhance procedural safety and tactile feedback during simulated endoscopic navigation and motivate further evaluation in clinically relevant settings in the future.

**Fig. 5.**
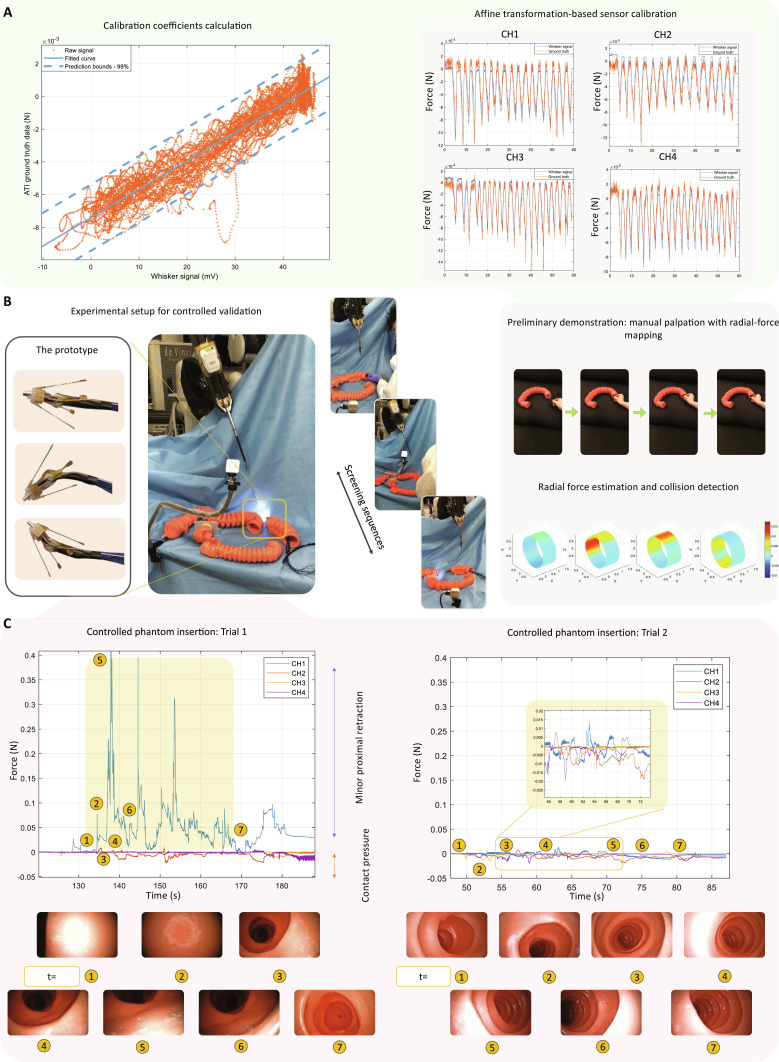
Radial force estimation and collision detection using the whisker sensor. (A) Calibration and channel-wise force fitting. (B) Handheld endoscopic simulation with colon phantom, and cylindrical force maps showing localized contact and pressure distribution. (C) Simulated endoscopic insertion using the Type II mount. Four-channel whisker force responses are shown with synchronized endoscopic views for Trial 1 and Trial 2.

The experimental procedure began with a calibration phase, essential for ensuring accurate force measurements across all whisker channels. Using sensor mount Type II, which features an outer diameter of 15 mm and a central through-hole with a 7-mm inner diameter in the current prototype design, we applied the affine transformation method again to calibrate each of the 4 sensing channels independently. In this setup, each whisker shaft is angled at 20° relative to the sensor mount axis, with a shaft length of 40 mm. The calibration process is illustrated in the upper left corner of Fig. 5A, showing the fitting process for Channel 3, where the raw whisker signal was aligned with ground-truth force data measured by an ATI Industrial Automation force sensor (ATI Industrial Automation, Inc., Apex, NC, USA). The upper right section of Fig. 5A provides a detailed view of the fitting performance, demonstrating how the calibrated whisker response closely aligns with the force sensor’s measurements.

Quantitative force-estimation performance is summarized in Table 1. Channel-wise calibration showed strong agreement with the ATI reference, and this performance was maintained in an independent validation dataset acquired under the same calibration configuration. Across CH1 to CH4, validation error remained at the millinewton level and correlation with the ATI reference remained high, supporting consistent force-estimation performance beyond the fitting set.

**Table 1.** Calibration and validation metrics for channel-wise radial force estimation. Channel-wise results for CH1 to CH4 are reported as calibration-set SSE, RMSE, and adjusted *R*^2^, together with validation-set RMSE, MAE, bias, and Pearson correlation relative to the ATI force-sensor reference, demonstrating reliable fit quality and consistent force-estimation performance across channels.

Source	Calibration			Validation			
	SSE	RMSE	Adjusted *R*^2^	RMSE	MAE	Bias	Pearson
CH1	0.0172	0.0009	0.9087	0.0012	0.0010	−0.0010	0.9724
CH2	0.0105	0.0008	0.9160	0.0021	0.0017	0.0017	0.9162
CH3	0.0257	0.0011	0.8991	0.0013	0.0010	0.0008	0.9487
CH4	0.0073	0.0007	0.9373	0.0009	0.0007	−0.0004	0.9510

With calibration completed, we first conducted a preliminary handheld demonstration to qualitatively validate the system’s capability for radial force mapping. As shown in the right-hand panel of Fig. 5B, the whisker system was manually guided along a colon phantom surface, mimicking the operational environment of endoluminal screening. During this exploratory palpation process, the system successfully recorded localized radial force responses, which were subsequently mapped onto a cylindrical model (right of Fig. 5B), visually capturing spatial force distributions. This preliminary trial not only confirmed the sensor’s responsiveness to varying contact intensities but also served as a practical demonstration of the system’s potential for force-guided exploration.

Building upon this demonstration, we proceeded to integrate the whisker system into a simulated endoscopic platform. As shown in the left-hand panel of Fig. 5B, the whisker system was mounted onto the distal tip of a flexible endoscope platform and inserted into a commercial colon phantom, enabling repeatable, fully controlled insertion trials in a procedurally relevant endoluminal setting. Two representative trials were performed to evaluate system behavior during continuous insertion and contact events. In Trial 1 (Movie S1), the insertion trajectory was suboptimal, requiring occasional proximal retraction to navigate the anatomical curvature. This maneuver introduced transient whisker deflections, which were particularly pronounced when the whiskers were pushed backward against the colon wall and produced distinct positive-going force spikes in the recorded signal. These large-amplitude peaks in the time-series plot (left panel of Fig. 5C) correspond to moments of whisker shaft reversal, as visually confirmed by synchronized endoscopic video frames shown below. The annotated sequence captures critical events such as initial wall contact, subsequent pressurization, and minor retraction-induced flicks, providing a comprehensive depiction of the mechanical interaction process.

In contrast, Trial 2 (Movie S1) presented a smoother and more continuous insertion trajectory, as depicted in the right segment of Fig. 5C. Here, the force signals across all 4 channels remained relatively stable and low in amplitude, indicating consistent navigation without sudden whisker deformation. The synchronized endoscopic views confirm minimal wall collision, supporting the hypothesis that optimal insertion angle and orientation substantially mitigate undesirable mechanical interaction. A zoom-in inset highlights a representative stable period, illustrating the signal behavior during smooth insertion.

Together, these experiments demonstrate the whisker system’s ability to detect radial contact and identify dynamic collision events under simulated, procedurally representative conditions. The ability to distinguish between high-contact states (as in Trial 1) and smooth navigation (as in Trial 2) reflects the system’s high spatiotemporal resolution and sensitivity to mechanical interaction. Furthermore, the integration of endoscopic imagery with time-aligned tactile signals establishes a multimodal framework for interpreting tool–tissue interactions in real time. This capability shows potential for augmenting procedural awareness during endoluminal navigation, where tactile feedback remains largely absent in current clinical practice. Although the current system operates within a distal light-contact force regime of approximately 0 to 0.02 N, it achieves a worst-case noise-limited force detectability of 10.8 μN within this range under a conventional 3σ criterion, thereby supporting early contact detection and other tasks requiring fine tactile discrimination, including compliance-related analysis. Future work will explore task-specific range extension through whisker-shaft redesign and deeper integration with robotic surgical workflows toward force-aware navigation and autonomous screening.

## Discussion

The benchmark and phantom-based experiments in this study define a controlled validation framework for clinically motivated tactile primitives, establishing the ability of the proposed system to capture relevant tactile dynamic under well-defined conditions, and thereby providing a basis for later disease-correlated validation in more clinically representative settings. At the same time, the proposed whisker system should more broadly be understood as a local tactile sensing layer within a larger adaptive robotic framework, rather than as a stand-alone solution for navigation in complex unstructured luminal environments. In such settings, intelligent intervention will depend not only on high-fidelity local perception of contact, texture, geometry, and radial loading, but also on its integration with morphological adaptation, locomotion, and navigation control. Recent work on multimodal magnetic robots and shared navigation strategies further suggests that robust operation in confined, heterogeneous, and dynamically changing environments requires coordinated adaptation across sensing, physical configuration, and control architecture [49–51]. Within this broader context, whisker-based tactile sensing could provide a complementary local observability channel for force-aware navigation and contact-informed interaction, while overall system-level adaptability would still depend on its coupling to robot mechanics and decision-making. Extending the present platform in this direction will require tighter integration between tactile sensing, mechanical compliance, and navigation control, so that local sensing can contribute to more robust endoluminal intervention in anatomically complex and unstructured environments.

### Whisker shaft property evaluation and fabrication

In the current design, acupuncture needles are utilized as the whisker shaft. Moving forward, our objective is to systematically evaluate the quantitative impacts of shaft characteristics, including diameter, material composition, length, and other related factors, on clinical performance, aiming to develop more optimal designs for enhanced performance. Numerous studies have shown that whisker geometry strongly affects dynamic behavior. In the present system, the assembled whisker sensors exhibited a dominant resonance of 69.24 Hz ± 1.89 Hz across the 4 channels, providing a useful descriptor of the dynamic operating regime of the current shaft–sensor assembly. In the present experiments, the task-relevant information remained well below this regime, while the texture benchmark, although the most frequency-sensitive of the three, also operated within a lower-frequency range under the scan conditions used. As highlighted in our previous modeling analysis, when approximating the whisker as a cantilever beam, the natural frequency f of this cylindrical beam depends on its material properties, geometry, and mass distribution, as indicated in Eq. (18).

$$f=\frac{\lambda_{1}^{2}}{2\pi L^{2}}\sqrt{\frac{\mathrm{EI}}{\mu }}$$ (18)

where $\lambda_{1}$ is 1.875, which is the first eigenvalue for a cantilever beam. I and μ are area moment of inertia and mass per unit length respectively, as shown in Eq. (19):

$$I=\frac{\pi d^{4}}{64}\;\mu =\rho A=\rho \left(\frac{\pi d^{2}}{4}\right)$$ (19)

The theoretical model assumes a perfectly rigid, fixed support; however, in practical fabrication, adhesive layers between structural elements introduce compliance at the support, effectively reducing the system’s stiffness and, consequently, its natural frequency. Furthermore, the added mass near the support from the strain gauge film increases system inertia, further decreasing the natural frequency. To broaden the whisker sensing potential across various application scenarios, finite element analysis will be taken into consideration to comprehensively evaluate complex geometries, material properties, and boundary conditions, with the aim of identifying improved design configurations.

### Whisker sensor mount design

The current configuration of the whisker sensor mount is shown in Fig. 2B. In particular, the present Type II mount has an outer diameter of 15 mm, which remains larger than desirable for routine integration with standard GI endoscopes. This limitation is primarily driven by the footprint of the individual whisker sensor units, especially the transducer component, together with the spatial requirements of the current Type II arrangement. Future iterations will therefore focus on miniaturizing the sensing unit itself, particularly through refinement of the transducer film, more compact packaging, and fabrication strategies based on microelectromechanical systems (MEMS) that could enable custom designed strain gauge geometries and a substantially smaller transducer footprint. In parallel, task-specific optimization could be achieved through a series of modular sensor mounts tailored to different procedural requirements. Alternatively, by leveraging mechanical design strategies, a reconfigurable mount could be developed to adjust the spatial distribution of the whiskers within a single platform, thereby enabling adaptation to different tactile sensing tasks without requiring a separate mount for each use case. Such task-specific configurability may also support distinct operational modes in future endoluminal use, with navigation-oriented configurations prioritizing radial contact monitoring during advancement and structure-sensitive configurations enabling controlled short-range interrogation of visually ambiguous local regions for subtle topographic or textural deviation.

### The actuation method during the experiments

The calibration of the KUKA robotic arm presents notable challenges, as leveling errors of the installation plane and multijoint kinematic inaccuracies can introduce low-frequency baseline fluctuations during the sweeping stage of the experiments. In principle, during this stage, the low-frequency baseline is expected to remain relatively stable as the whisker tip traverses the test surface. In practice, however, robotic actuation introduced slow drift components superimposed on the texture-related response. To reduce this effect, each sweep segment was first corrected by removing a dominant constant baseline offset from the stable precontact portion of the recording, after which LOESS was applied independently to each channel to estimate the slowly varying baseline trend. Subtracting this trend from the original sweep segment preserved the texture-related AC component used for the time domain, frequency domain, and time–frequency analyses. Notably, experimental results obtained with handheld operation of the whisker system demonstrate limited impact of the actuation mode on the texture-related signal, as distinct textures from different test objects remained clearly distinguishable, underscoring the high sensitivity of the proposed system to texture features.

In shape reconstruction tasks, these calibration challenges introduce additional complexity. The present proof-of-concept demonstrates local geometric sensing under controlled scanning, but it does not yet include an independent localization. Accordingly, the reconstructed *X*-axis in Fig. 4 depends on the motion of the driving platform rather than on direct positional measurement. In a future clinically relevant endoluminal setting, we envisage this mode primarily as a short-range, controlled scanning function for interrogating visually ambiguous local regions, where whisker-based tactile sensing would complement vision rather than replace it. In that setting, effective local 3D reconstruction would require an additional mechanism for relative displacement or pose estimation alongside the whisker signals. This could be achieved through integration with positional tracking modalities such as electromagnetic tracking [52], optical or vision-based localization [53], fiber-based shape sensing [54], or hybrid combinations of these approaches. In addition, if implemented on a robotic continuum or tendon-driven platform, relative displacement estimation could also be supported by robot-state reconstruction from actuation-space information, for example, tendon displacement combined with embedded curvature or strain sensing [55,56] to improve tip-pose estimation. Within the present configuration, the system supports local relative geometric reconstruction under controlled scanning conditions.

Another important factor is the orientation angle of the whisker system. Experimental observations reveal that the angle at which the whisker tip approaches the test object, the system’s inclination relative to the object, significantly impacts scanning outcomes. When the system is oriented perpendicular to the test surface, sensor sensitivity increases, leading to larger deformation and thus greater sensor responsiveness. However, this orientation also leads to a “flick out” effect in subsequent scanning stages after reaching the highest point of the tested subjects, limiting effective contour tracking. Adjusting the inclination angle substantially mitigates this issue; in our experiments, a tilt angle of approximately 25° between the whisker shaft and the horizontal plane was used, yielding high-resolution results. Future studies will explore adaptive angle adjustments to balance SNR and screening performance.

Beyond calibration considerations, phantom-based studies warrant mention. Concerning the effectiveness of phantom-based experiments, despite substantial differences between phantoms and biological tissues, the phantom-based approach remains important in numerous clinically oriented studies. In the future, transitioning from phantom-based data to a patient database will require algorithmic fine-tuning to optimize performance, enhancing the potential of the whisker modality for clinical applications.

### The calibration method for the proposed whisker system

In this work, we adopted an affine transformation-based calibration framework, enhanced with robust fitting, to reduce interchannel inconsistency arising from manufacturing tolerances, installation variability, and accumulated gain differences across the signal-conditioning chain. A principal advantage of this approach is that it remains computationally lightweight, interpretable, and readily deployable in real time, while still providing effective correction of channel-wise sensitivity and offset mismatch. Within the present hardware configuration, this was sufficient to improve cross-channel consistency for downstream tasks such as shape reconstruction and radial force estimation.

In the present system, the affine transformation is used primarily to compensate interchannel gain and offset mismatch under a fixed experimental configuration, thereby improving cross-channel consistency and measurement interpretability with low computational overhead. Its coefficients, however, are not universal system constants. Their transferability depends on the stability of the sensing assembly, mounting geometry, and whisker condition, and substantial changes in these factors may require recalibration.

At the same time, the method has clear limitations that should be acknowledged. It does not explicitly represent hysteresis, load-dependent coupling, posture-dependent contact variation, or temporal drift outside the calibration condition. Severe slipping, fold-over, or strongly posture-dependent contact would place the system in a state-dependent interaction regime for which a single affine correction is unlikely to remain valid. In the current study, we did not perform a dedicated evaluation that systematically isolated these strongly nonlinear interaction states and therefore do not yet have a sufficiently strong basis for reporting a reliable quantitative error range for them. In broader intended endoluminal use, part of the design strategy will also be to reduce the occurrence of such unstable contact states through task-specific mechanical optimization, for example, by adjusting whisker length, shaft stiffness, and contact orientation. Future clinically representative evaluation should therefore examine calibration robustness under slipping-, bending-, and fold-related nonlinear contacts using conventional error metrics together with hysteresis and repeatability analysis under repeated or direction-reversed scans. As operating conditions broaden and interchannel behavior becomes more nonlinear, more flexible calibration models, including data-driven approaches, may become advantageous not only for moving beyond channel-level calibration alone, but also for supporting task-level decision making in applications such as navigation assistance, contact interpretation, and lesion-related sensing.

### Translational considerations and clinical pathway

The compact design of the system is important at both the distal sensing interface and the signal-conditioning circuit level. From a translational perspective, further reduction of the distal mount dimensions will be necessary before the system can be considered for routine integration with standard GI endoscopes. In addition to miniaturizing the sensor mount itself, integrating a more compact signal-conditioning circuit closer to the sensing unit could reduce interconnect length and further improve the SNR. For future in vivo or clinically deployed implementations, however, this miniaturization pathway must be considered together with material safety and reprocessing requirements. The present prototype has not yet undergone formal biocompatibility evaluation, and future translational development will require either appropriate selection of tissue-contacting distal components, including the whisker sensing elements and mount surfaces, or validated biocompatible encapsulation and barrier layers that isolate internal transduction layers, adhesives, and electrical interconnects from the mucosal environment. These design constraints also impose important requirements on system architecture. In particular, the choice between a single-use distal add-on and a reusable mount carries different design implications. A single-use distal add-on could reduce the burden of reprocessing at the tissue-contacting interface by allowing the distal whisker sensing structure to be discarded after use. By contrast, a reusable configuration would require the tissue-contacting distal sensing structure to tolerate repeated sterilization and reprocessing without unacceptable degradation in sensing performance. Under such a design, sterilization and reprocessing would need to be evaluated not only for biocompatibility and encapsulation integrity, but also for their effects on whisker stiffness, adhesive stability, baseline drift, and calibration consistency, as each of these factors could directly influence sensing performance.

These engineering and safety considerations also define the practical sequence of steps required for clinical translation. Following further benchtop refinement of miniaturization, sensing stability, and packaging, the next stage will be evaluation in progressively more realistic experimental settings, including ex vivo tissue models and controlled in vivo studies, to assess sensing performance under physiological contact conditions, luminal motion, fluid exposure, and biologically relevant tissue variability. This will be particularly important for determining how the whisker system behaves in slippery, compliant, and mucus-rich environments, where tissue response is influenced not only by surface geometry but also by viscoelasticity, hysteresis, stress relaxation, creep, and lubrication-mediated contact effects. These factors can alter both texture-related tactile signatures and the force–deformation relationship during interaction, and may therefore affect tactile feature separability, calibration stability, and force-readout reliability. Beyond technical validation alone, translational assessment will also require workflow-oriented studies to determine how tactile outputs are interpreted by operators or robotic control systems during navigation and lesion assessment. From a regulatory perspective, future development will need to incorporate device-grade verification of biocompatibility, electrical safety, sterilization compatibility, packaging integrity, and manufacturing consistency, together with risk management appropriate for a patient-contacting endoluminal add-on device. Addressing these requirements will be important for advancing the present proof-of-concept system toward clinically in vivo deployment.

## Conclusion

In this study, a bionic multichannel whisker system is designed to address the limitations of conventional endoscopic tools in sensing tissue properties during endoluminal interventions. Inspired by the tactile capabilities of natural whiskers, the system integrates advanced hardware and signal processing techniques, including strain gauge-based sensors, affine transformation calibration, and modular designs to provide a multisensing system (capable of assessing surface topology, surface texture, and radial force) in a compact form factor. Experimental validation demonstrates the system’s robustness in texture discrimination, shape reconstruction, and radial force estimation, suggesting its potential to augment visual inspection with quantitative tactile feedback during endoluminal procedures.

The adoption of affine transformation for calibration not only ensures cross-channel consistency but also mitigates the effects of manufacturing variances and environmental noise, establishing a reliable framework for tactile sensing. Moreover, the compact and modular design of the whisker system has the potential to enable seamless integration with existing endoscopic platforms while maintaining versatility for diverse clinical applications.

Future work aims to further improve spatial and temporal resolution, expand the system’s sensing capabilities through deep learning-based calibration, and explore its integration into large-scale medical databases for enhanced clinical decision-making. The proposed system represents a important step toward next-generation surgical robotics, paving the way for safe, precise, and reliable endoluminal diagnostics.

## Data Availability

All data needed to evaluate the conclusions in the paper are present in the paper and/or the Supplementary Materials, and the datasets during and/or analyzed during the current study are available from the corresponding authors upon reasonable request.
