# Supplementary material for: A Bionic Multichannel Whisker System for Assisting Endoluminal Intervention
Source: Cyborg Bionic Syst. 2026 Aug 5;7:0616. doi: 10.34133/cbsystems.0616 (PMC13438030; doi:10.34133/cbsystems.0616)
Supplement: Supplementary file 1 [file cbsystems.0616.f1.docx]

Supplementary Material

Title

A Bionic Multichannel Whisker System for Assisting Endoluminal Intervention

**Authors**

Zeyu Wang^1^, Frank P.-W. Lo^1^, Junhong Chen^1^, Jianing Qiu^2^, Qian Chen^1^, Yongqi Zhang^1^, Chen Lin^1^, Kyle Lam^1^, James Calo^1^, Benny P.L. Lo^1*^†, Alexander J. Thompson^1*^†, Eric M. Yeatman^1*^†

**This PDF file includes:**

Supplementary Methods 1 and 2

Figures. S1 to S7

Tables S1 and S2.

**Other Supplementary Materials for this manuscript include the following:**

Movie S1. Endoscopic view during Trial 01, recorded synchronously with whisker sensing, illustrating instrument–tissue interaction during endoluminal navigation.

Movie S2. Endoscopic view during Trial 02 (as in Movie S1).

**Method 1. Extraction of texture related AC components**

To isolate the texture-related dynamic response from low frequency baseline fluctuations introduced by robotic actuation, each multichannel whisker recording was processed in two stages. A constant baseline offset was first removed, followed by segment wise detrending using locally estimated scatterplot smoothing (LOESS). The procedure was applied independently to each of the four sensing channels and to each sweep stage segment used for texture analysis.

Let $x_{c}[n]$ denote the raw discrete time signal from channel $c\in\{1,2,3,4\}$, where $n$ is the sample index. A constant baseline correction was first applied using a reference sample acquired during the pre-contact baseline period, yielding

$$\tilde{x}_{c}\left[ n \right]=x_{c}\left[ n \right]-x_{c}\left[ n_{0} \right]. (S1)$$

Here, $n_{0}$ denotes a baseline reference sample selected from the stable pre-contact portion of the recording. Sweep stage segments were then extracted from the corrected signal and denoted by

$$s_{k,c}\left[ m \right]=\tilde{x}_{c}\left[ n_{k,\mathrm{start}}+m-1 \right],m=1,\ldots,N_{k}, (S2)$$

where $k\in\{1,\ldots,5\}$indexes the five texture conditions, $\left[ n_{k,\mathrm{start}},n_{k,\mathrm{end}} \right]$defines the sweep interval for condition $k$, and $N_{k}=n_{k,\mathrm{end}}-n_{k,\mathrm{start}}+1$. These intervals correspond to the stable sweeping portions of the whisker–surface interaction traces used for downstream analysis.

For each segment and channel, the slowly varying baseline trend was estimated using a LOESS smoother with span parameter $\alpha=0.2$,

$$\hat{b}_{k,c}\left[ m \right]=\mathcal{L}_{\alpha}\left\{ s_{k,c}\left[ m \right] \right\},\alpha=0.2, (S3)$$

where $\mathcal{L}_{\alpha}(\cdot)$denotes the LOESS smoothing operator. The detrended texture related AC component was then obtained as

$$y_{k,c}\left[ m \right]=s_{k,c}\left[ m \right]-\hat{b}_{k,c}\left[ m \right]. (S4)$$

This operation suppresses low frequency drift while preserving the faster fluctuations associated with whisker–surface interaction during the sweep stage. The resulting signal $y_{k,c}[m]$ was used for subsequent time domain visualization, power spectral density estimation, and time frequency analysis.

**Method 2. Implementation of the multichannel signal-conditioning hardware**

The signal-conditioning hardware was implemented as a stacked architecture comprising a front-end preprocessing board and a backend acquisition board, consistent with the block partition and embedded acquisition flow shown in Figs. S3–S4. The preprocessing module was realized on a 2-layer PCB and interfaced directly with the strain-gauge bridge outputs for first-stage analogue conditioning. The backend subsystem, integrating the analogue front end (AFE), microcontroller unit (MCU), data interface, and power-management circuitry, was implemented on a 4-layer high-density interconnect (HDI) PCB. In this layout, L1 and L4 were signal layers, L2 was a ground plane with analogue–digital isolation, and L3 was the power plane.

Multichannel acquisition was organized around an AFE-based synchronous readout framework with MCU-controlled timing and DRDY-triggered data retrieval through the serial peripheral interface (SPI), enabling stable channel alignment and configurable acquisition control. The present power architecture used two cascaded low-dropout regulators (LDOs) to provide a low-noise supply for the analogue and mixed-signal stages, with the known trade-off of reduced power efficiency. Future iterations could adopt a switched-mode direct current-to-direct current converter (DC–DC converter) combined with LDO regulation to better balance efficiency and noise performance.

The PCBs were designed in Altium Designer 2021 (Altium LLC, San Diego, CA, USA), the structural housing was developed in SolidWorks 2020 (Dassault Systèmes SolidWorks Corporation, Waltham, MA, USA), and prototype mechanical parts were fabricated using an Objet500 Connex3 3D Printer (Stratasys Ltd., Eden Prairie, MN, USA). These implementation details are provided here to improve reproducibility while keeping the main text focused on system architecture and experimental validation.

**Fig. S1. Analytical modeling of the whisker–environment interaction:** static (top) configuration: the whisker is treated as a cantilevered beam under quasi-static loading, relating localized transverse force to deflection and slope along the shaft; dynamic (bottom) configuration: base actuation and time-dependent deformation represent active whisking, enabling characterization of transient contacts and spatial force distribution during continuous exploration.

**Fig. S2. Distal tip treatment of the whisker shaft in the current prototype.** The distal end of each acupuncture-needle whisker was shaped and covered with a polyimide film to soften the terminal contact region and provide a degree of protection during interaction with the lumen wall.

**Fig. S3. Signal conditioning circuit for multichannel whisker acquisition.** The compact PCB integrates analogue input channels, power management, an analogue front end, an MCU subsystem, and a BLE wireless subsystem, while retaining wired communication for data transfer and system interfacing.

**Fig. S4. Embedded control flow for power management and ADC acquisition in the multi-channel whisker system.** The sequence proceeds from the charge submodule and Li-ion battery unit to multi-rail power generation and ADC clock selection using internal or external sources to balance power consumption and timing stability. A reset pulse initializes the digital logic, followed by register reset and reference configuration. The acquisition stage enables the reference submodule, starts conversion, and issues commands continuous streaming. DRDY asserts data readiness and triggers an interrupt service routine for deterministic readout and data transmission, with a waiting state between DRDY events.

**Fig. S5. Sandpaper substrates for texture discrimination using varying grit sizes (P80 to P2000).**

**Fig. S6. Additional shape-reconstruction results for the tilted benchmark set.** Top row: benchmark objects with tilted stepped features. Middle row: STL-derived ground-truth surfaces. Bottom row: calibrated reconstructed results obtained with the proposed whisker system.

**Fig. S7. Radial force calibration using a Nano17 force sensor (ATI Industrial Automation, USA).** The whisker system was actuated against the reference sensor to obtain ground truth force measurements. Time synchronized whisker outputs and Nano17 readings were used to fit the radial force mapping and quantify calibration error across the tested force range.

**Table S1. Comparison of representative tactile sensing approaches relevant to minimally invasive interventions**. Included works are summarized by clinical context and sensing location, together with the sensing principle, inferred/measured interaction variables, and the key characteristics and limitations of each solution.

| Works | Clinical context | Sensing location | Sensing principles | Inferred variables and contributions | Characteristics and main limitation for endoluminal use |
| --- | --- | --- | --- | --- | --- |
| Peirs et al [1]  (2004) | MIS / robotic surgery | Distal instrument tip (5 mm) | Titanium flexure with optical-fiber intensity readout | Estimates tri-axial force; achieved design ranges 2.5N axial / 1.7N radial; | Pros: Small distal footprint; electrically safe optical sensing head;  Limits: Sensitivity/resolution limited by fabrication and alignment; optical intensity sensing requires calibration; fiber routing constraints |
| Lee et al [2]  (2019) | General robotics / prosthetics e-skin | Large-area skin-like arrays | Event-based tactile communication architecture for multimodal distributed sensors | Distributed pressure / slip / texture-related spatiotemporal patterns | Pros: Rich spatial contact information; scalable multiplexed sensing concept  Limits: Not tool-native for slender endoluminal integration; requires distributed electronics/power per node and a nontrivial decoder |
| Kim et al [3]  (2011) | Cardiac EP mapping and RF ablation | Conformal balloon surface | Stretchable tactile and thermal sensor mesh | Contact state / pressure distribution / temperature | Pros: Conformal multimodal sensing on curved soft tissue.  Limits: Primarily normal-pressure/contact sensing; limited property-specific inference; fabrication and integration complexity |
| Oh et al [4]  (2020) | Robotic manipulation / closed-loop grip control | Flexible fingertip array | Active-matrix dual-gate ZnO piezoelectric TFT array | High-density tactile image; pressure / slip-related information | Pros: High spatial resolution; multiplexed tactile imaging  Limits: mechanically mediated cross-talk/force spreading can affect spatial fidelity; array-level integration, routing, and packaging remain difficult in very slender fluid-exposed tools |
| Hou et al [5]  (2024) | Flexible endoscopy / NOTES-style manipulation | Distal endoscopic forceps jaw | Piezoresistive MEMS sensor with force-transfer layer | Local normal and shear force with calibrated ranges ±0.6 N (shear) and 0–1.2 N (normal) | Pros: Very small distal form factor; fast response; direct force sensing at contact site  Limits: Primarily local jaw-force measurement rather than distributed tactile perception including texture and shape perception; cross-axis effects and assembly sensitivity  still a local jaw force measurement |
| Zhang et al [6]  (2024) | Flexible endoscopy / luminal navigation | Distal endoscope tip | FBG-based soft contact sensor | Estimates contact force magnitude (0–1.5 N) and contact orientation | Pros: Endoscope-compatible integration; soft contact monitoring; real-time tactile–visual cueing  Limits: Mainly force/orientation feedback; requires optical interrogator; does not directly provide texture or local topography sensing |
| Gao et al [7]  (2025) | Cochlear implantation | Distal forceps tip | FBG arrays on a curved substrate | Enables 6-axis force/torque sensing at the tool tip | Pros: Multi-axis distal force/torque measurement in compact form  Limits: Requires fiber routing and interrogator; calibration complexity; local F/T sensing rather than broader tactile surface interrogation measures |
| Cui et al [8]  (2026) | Tactile endoscopy / in vivo diagnosis | Distal 4.8–5 mm endoscopic probe integrated at the tip | Vision-based deformation inference with integrated thermal sensing | 3D contact force and temperature distribution | Pros: Reuses image-like information; compact multimodal tip-level sensing.  Limits: force is inference-driven (requires training, compute, and calibration), and marker coatings occlude FOV (needs inpainting) |
| This study | Endoluminal intervention / proof-of-concept whisker add-on | Distal add-on whisker module | Multichannel whisker sensing with strain-gauge transduction and shared biomimetic architecture | Fine surface irregularity, millimeter-scale local geometric variation, and early radial contact awareness | Pros: Supports multiple tactile primitives within one compact add-on sensing framework; complementary to vision-centric endoscopy  Limits: Proof-of-concept stage; not a replacement for all force-sensing methods; further miniaturization and clinically representative validation remain required |

[1]	J. Peirs, J. Clijnen, D. Reynaerts, H. V. Brussel, P. Herijgers, B. Corteville, and S. Boone, “A micro optical force sensor for force feedback during minimally invasive robotic surgery,” Sensors and Actuators A: Physical, vol. 115, no. 2, pp. 447–455, Sept. 2004.

[2]	W. W. Lee, Y. J. Tan, H. Yao, S. Li, H. H. See, M. Hon, K. A. Ng, B. Xiong, J. S. Ho, and B. C. K. Tee, “A neuro-inspired artificial peripheral nervous system for scalable electronic skins,” Science Robotics, vol. 4, no. 32, p. eaax2198, July 2019.

[3]	D.-H. Kim, N. Lu, R. Ghaffari, Y.-S. Kim, S. P. Lee, L. Xu, J. Wu, R.-H. Kim, J. Song, Z. Liu, J. Viventi, B. de Graff, B. Elolampi, M. Mansour, M. J. Slepian, S. Hwang, J. D. Moss, S.-M. Won, Y. Huang, B. Litt, and J. A. Rogers, “Materials for multifunctional balloon catheters with capabilities in cardiac electrophysiological mapping and ablation therapy,” Nature Mater, vol. 10, no. 4, pp. 316–323, Apr. 2011.

[4]	H. Oh, G.-C. Yi, M. Yip, and S. A. Dayeh, “Scalable tactile sensor arrays on flexible substrates with high spatiotemporal resolution enabling slip and grip for closed-loop robotics,” Science Advances, vol. 6, no. 46, p. eabd7795, Nov. 2020.

[5]	C. Hou, H. Gao, X. Yang, G. Xue, X. Zuo, Y. Li, D. Li, B. Lu, H. Ren, H. Liu, and L. Sun, “A piezoresistive-based 3-axial MEMS tactile sensor and integrated surgical forceps for gastrointestinal endoscopic minimally invasive surgery,” Microsyst Nanoeng, vol. 10, no. 1, p. 141, Sept. 2024.

[6]	Y. Zhang, Q. Jiang, F. Wang, J. Wang, M. Ye, and D. Li, “Tac-scope: An endoscope integrated with soft sensor for real-time tactile feedback,” Measurement, vol. 235, p. 114953, Aug. 2024.

[7]	H. Gao, H. Liu, H. Jia, Z. Lin, Y. Zou, Z. Xu, S. Huang, H. Tan, H. Wu, W. Chen, and A. Gao, “Multi-axis robotic forceps with decoupled pneumatic actuation and force sensing for cochlear implantation,” Nat Commun, vol. 16, no. 1, p. 1648, Feb. 2025.

[8]	S. Cui, L. Li, Z.-X. Huang, Y. Yu, M. Cai, X. Bao, C. Zhang, T. Zhang, L. Cheng, W. Zhang, Z. Lou, S. Wang, W. Gong, C.-F. Wu, L. Wang, and Y. Wang, “Superelastic Tellurium Thermoelectric Coatings for Advanced Trimodal Microsensing,” Nat Commun, Jan. 2026.

| Sample ID | Set | Geometry | State | Pearson | Spearman | SSIM | Cosine similarity | MAE z (mm) |
| --- | --- | --- | --- | --- | --- | --- | --- | --- |
| 01 | I | Wavy | Uncalibrated | 0.2781 | 0.1002 | 0.2784 | 0.2781 | 2.5005 |
|  | I |  | Calibrated | 0.9302 | 0.8703 | 0.9302 | 0.9302 | 0.5250 |
| 02 | I | Rectangular | Uncalibrated | 0.1417 | -0.0178 | 0.1421 | 0.1417 | 3.1090 |
|  | I |  | Calibrated | 0.7361 | 0.5992 | 0.7362 | 0.7361 | 1.2724 |
| 03 | I | Triangular | Uncalibrated | -0.0476 | -0.1671 | -0.0471 | -0.0476 | 2.2339 |
|  | I |  | Calibrated | 0.7486 | 0.6168 | 0.7487 | 0.7486 | 0.9918 |
| 04 | II | Wavy tilted | Uncalibrated | -0.1366 | -0.1876 | -0.1361 | -0.1366 | 1.6692 |
|  | II |  | Calibrated | 0.8919 | 0.6302 | 0.8919 | 0.8919 | 0.5280 |
| 05 | II | Rectangular tilted | Uncalibrated | 0.2373 | 0.0044 | 0.2377 | 0.2373 | 1.8932 |
|  | II |  | Calibrated | 0.7624 | 0.5388 | 0.7625 | 0.7624 | 0.6125 |
| 06 | II | Triangular tilted | Uncalibrated | 0.0117 | -0.0618 | 0.0123 | 0.0117 | 1.8976 |
|  | II |  | Calibrated | 0.7059 | 0.5229 | 0.7060 | 0.7059 | 0.9380 |

**Table S2. Quantitative evaluation of shape reconstruction under uncalibrated and calibrated conditions.** Reconstruction fidelity against the STL-derived local ground-truth strip is reported using Pearson correlation, Spearman correlation, structural similarity index measure, cosine similarity, and MAE in Z axis. Calibrated results correspond to the final reconstruction after applying the fixed correction determined from the reference sample.
